# Causal relationship between atrial fibrillation/warfarin and cutaneous melanoma: a two-sample Mendelian randomization study

**DOI:** 10.3389/fmed.2024.1336849

**Published:** 2024-03-05

**Authors:** Wang Xiaowu, Zhou Qiang, Han Yike, Zhixuan Wu, Jin Yiheng, Chen Xuemei, Lin Sen, Chen Jiong

**Affiliations:** ^1^Department of Burns and Skin Repair Surgery, The Third Affiliated Hospital of Wenzhou Medical University (Ruian People’s Hospital), Wenzhou, China; ^2^Department of Otolaryngology, The Third Affiliated Hospital of Wenzhou Medical University (Ruian People’s Hospital), Wenzhou, China; ^3^Department of Gynaecology and Obstetrics, The Third Affiliated Hospital of Wenzhou Medical University (Ruian People’s Hospital), Wenzhou, China; ^4^Department of Nail and Breast Surgery, The First Affiliated Hospital of Wenzhou Medical University, Wenzhou, China; ^5^College of Public Health and Management, Wenzhou Medical University, Wenzhou, China; ^6^School of Pharmaceutical Sciences, Wenzhou Medical University, Wenzhou, China

**Keywords:** atrial fibrillation, warfarin, cutaneous melanoma, Mendelian randomization study, sensitivity analysis

## Abstract

**Purpose:**

In recent years, the relationship between malignant tumors and atrial fibrillation has attracted more and more attention. Atrial fibrillation can also cause a series of adverse events, such as the risk of thromboembolism. Also, Warfarin is often used here. But, the relationship between cutaneous melanoma and atrial fibrillation, and between cutaneous melanoma and warfarin is still unclear. Therefore, we used a two-sample Mendelian randomization to assess the causal relationship between atrial fibrillation/warfarin and cutaneous melanoma (cM).

**Methods:**

Firstly, atrial fibrillation (ukb-b-11550; *n*Case = 3,518, *n*Control = 459,415) and warfarin (ukb-b-13248; *n*Case = 4,623, *n*Control = 458,310) as exposures, with genome-wide association studies (GWAS) data from the United Kingdom Biobank. And cM (ieu-b-4969; *n*Case = 3,751, *n*Control = 372,016) as outcome, with GWAS data from the IEU Open GWAS project. Subsequently, single-nucleotide polymorphisms (SNPs) were filtered from GWAS studies using quality control measures. In addition, two-sample Mendelian randomization (MR) analysis was performed to explore the causal relationship between atrial fibrillation or warfarin and cM and used inverse variance weighting (IVW) as the primary analytical method. Finally, relevant heterogeneity and sensitivity analysis were performed to ensure the accuracy of the results.

**Results:**

A causal relationship between atrial fibrillation and cutaneous melanoma was observed, and between warfarin and cutaneous melanoma.

**Conclusion:**

The atrial fibrillation may play a causal role in the development of cutaneous melanoma, but the mechanism and the causal relationship between warfarin and cutaneous melanoma needs to be further elucidated.

## 1 Introduction

Cutaneous melanoma (cM) develops through the malignant transformation of melanocytes in the basal layer of the epidermis under the influence of various factors. Genetic factors and UV radiation are the main contributors to cM (1). The global incidence of cM is approximately 160,000 new cases per year, resulting in 48,000 deaths (2). In recent years, the incidence of cM is increasing all over the world, surpassing that of any other solid tumor (2, 3), especially in most European countries. Queensland has the highest recorded incidence of cM among them. As for the treatment for primary cM involves surgery and lymph node dissection, while immunotherapy and targeted therapy are the main approaches for advanced metastatic cM (4). However, there are still limitations in the treatment of advanced cM. So, preventing and treating advanced cM are very important.

In recent years, the relationship between malignant tumors and atrial fibrillation has attracted more and more attention (5). Many literatures reported that the incidence of new atrial fibrillation in patients with malignant tumors has increased (6, 7). Meanwhile, in patients with atrial fibrillation, the prevalence of malignant tumors also increased (8). At present, the pathophysiological mechanism of atrial fibrillation in patients with malignant tumors is not completely clear. It is generally believed that adverse internal environment (electrolyte disorder, metabolic disorder, inflammatory reaction, etc.), autonomic nervous dysfunction caused by cancer pain or depression, myocardial dysfunction caused by anti-tumor related chemotherapy, and other adverse factors are all inducing factors of atrial fibrillation (5). With the increasing incidence of cM (9) and the global population aging (10), the number of cM patients with atrial fibrillation is increasing gradually (11). The treatment and management of these patients require more attention and response. However, the relationship between atrial fibrillation and cM remains unclear. Mincu et al. (12) found that patients with cM will have related cardiovascular adverse events, including atrial fibrillation when receiving targeted therapy and chemotherapy. However, once patients with cM are accompanied by atrial fibrillation, the prognosis is often poor. Moreover, atrial fibrillation can also cause a series of adverse events, such as the risk of thromboembolism (13). Therefore, it is inevitably used to prevent thromboembolism under the condition of meeting the no contraindication of using anticoagulants (warfarin represented) (14). However, the relationship between cM and warfarin is not clear. Currently, it is reported that warfarin is related to the proliferation, apoptosis, and migration of melanoma cells (15). In summary, there exists a certain relationship between cM and atrial fibrillation, or cM and warfarin, although the causal relationship remains unknown. Consequently, research in this area holds significant clinical significance.

Mendelian Randomization (MR) (16) is an approach to data analysis that is used to test etiological reasoning. In MR, to analyze the causal link between exposure variables and outcomes in epidemiological research, genetic variation instrumental variables closely related to exposure variables are utilized to replace exposure variables (17). Also, two-sample MR (TSMR) gathers exposure and outcome from two independent data sets and uses the MR method to assess the causal connection between them. The GWAS website is an open resource that may be utilized for obtaining disease outcomes and exposure variables on a global scale. Thus, TSMR and GWAS data set can be utilized to investigate the causal relationship between illness outcome and exposure variables (18).

In this work, we employed a TSMR approach design to look for a causal relationship between cM and atrial fibrillation, or cM and warfarin. We investigate the causal relationship between atrial fibrillation/ warfarin as an exposure, and cM as a result of outcome.

## 2 Materials and methods

### 2.1 Study design

**Figure 1:**
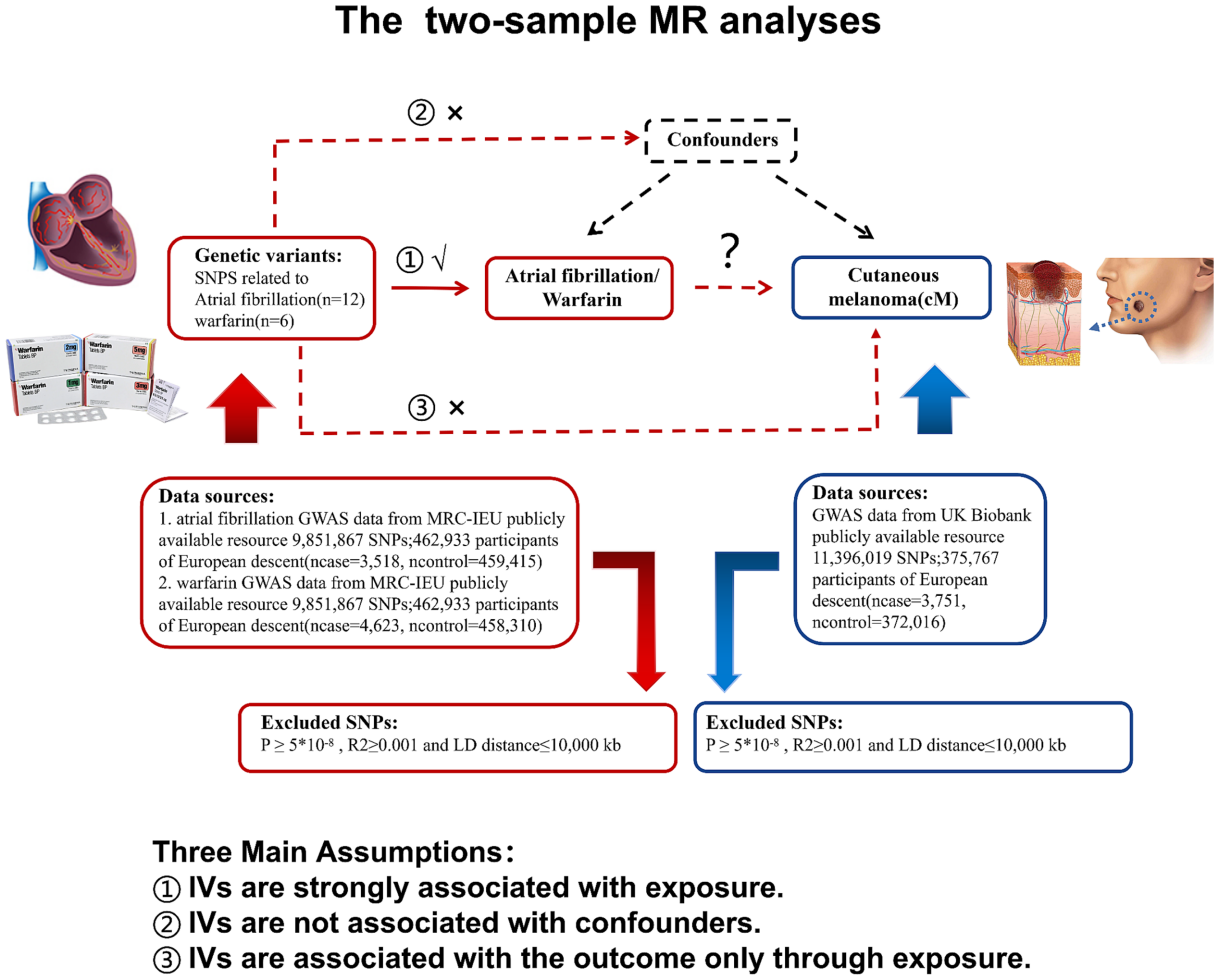
Research flow chart: atrial fibrillation or warfarin as exposure in two-sample MR analysis, marked in red; cutaneous melanoma as a result, marked in blue. MR, Mendelian randomization; LD, Linkage disequilibrium; and SNP, Single nucleotide polymorphism.

The purpose of this study is to investigate the causal relationship between cM and atrial fibrillation/warfarin. Therefore, the analysis method of TSMR study in this study is shown in Figure 1.

### 2.2 Data sources

Genome-wide association studies (GWAS) of cM, warfarin and atrial fibrillation are from the GWAS database^1^ (19). Specifically, the GWAS data set for cM is sourced from the United Kingdom Biobank,^2^ while the data set for warfarin is obtained from the MRC-IEU,^3^ and the data set for atrial fibrillation is acquired from the MRC-IEU. The participants included in these data sources are exclusively of European descent. This deliberate selection of European participants helps ensure a more homogeneous sample population, reducing confounding factors and enhancing the reliability of the results. The data included in this study has been published, so ethical approval or informed consent is not required. Finally, the details of the data sources are shown in Table 1.

**Table 1:** Warfarin, atrial fibrillation, and cutaneous melanoma summary data sources.

Exposure^*^/Outcome^#^	GWAS ID	Sample size	Number of SNPs	Population	Consortium	Sex	Year
Cutaneous melanoma (cM)/Melanoma skin cancer^#^	ieu-b-4969	375,767	11,396,019	European	United Kingdom Biobank	Males and females	2021
Warfarin^*^	ukb-b-13248	462,933	9,851,867	European	MRC-IEU	Males and females	2018
Atrial fibrillation^*^	ukb-b-11550	462,933	9,851,867	European	MRC-IEU	Males and females	2018

^*^Represents the exposure factor and ^#^represents the outcome factor.

### 2.3 Instrumental variables selection

In order to identify the instrumental variable (IV) for this investigation, we first searched the entire genome for single nucleotide polymorphisms (SNPs) under the following circumstances (19–21). The following circumstances are that (1) SNPs have a strong relationship with exposure (Warfarin/Atrial fibrillation); (2) Confounding variables that impact exposure (warfarin/atrial fibrillation) and outcome (cM) are unrelated to SNPs; and (3) SNPs only have an impact on outcome (cM) through the exposure (warfarin/atrial fibrillation) passway and are not directly connected to outcome (cM). Based on the above requirements, we used R (4.3.0) software to obtain respective SNPs with *p* < 5*10^−8^, the genetic distance of 10,000 KB, and linkage disequilibrium parameter *r*^2^ < 0.001 from cM, warfarin, and atrial fibrosis GWAS data sets. Then, we use PhenoScanner^4^ to find out whether SNPs contain known confounding, and if so, we will eliminate the SNP.

### 2.4 Two-sample Mendelian randomization analysis

Mendelian randomization analysis is a prominent tool for determining the causal effect of exposure variables on outcomes via genetic variation (18, 22). To confirm the causal association between atrial fibrillation/warfarin and cM, we used various MR techniques, including inverse variance weighted (IVW), MR-Egger, weighted median, simple mode, and weighted mode. The IVW and the MR-egger techniques are often employed as basic magnetic resonance methodologies in worldwide MR analysis. IVW is the primary tool for determining if there is a causal link between exposure variables and outcomes in MR analysis. When the *p* value of IVW is less than 0.05, the result is considered significant. Under the condition of IVW, the direction of MR-egger and the weighted median method must be the same as that of IVW. In addition, Cochran’s *Q* test (23), MR-egger regression (24), and MR-presso test (25) are used for sensitivity analysis to identify heterogeneity, evaluate pleiotropy, and correct level pleiotropy. Then, by removing one SNP test at a time, the final result is depicted by a forest diagram, scatter diagram, and funnel diagram. The aforementioned MR analysis methods are all implemented by the “TwoSampleMR” and “MRPRESSO” R packages of R language (version 4.3.0).

## 3 Results

### 3.1 Selection of instrumental variables between cM and atrial fibrillation, or cM and warfarin

We identified instrument SNPs with strong association qualities (*p* < 0.05) from the TSMR analysis of atrial fibrillation and cM. We discovered 14 SNPs (Supplementary Table S1). Following that, when we extracted the information of the instrumental in the result, we discovered one SNP that was excluded because there were no associated outcomes (rs1690117). When reconciling exposure and outcome data, one SNP (rs10821415) is removed since it is palindromic. We have 12 SNPs for MR analysis in the end. Similarly, we removed three SNPs without outcome information from the initial nine SNPs in the MR analysis of cM and warfarin (rs4656683, rs2060826, and rs4834327). Last, we had 18 SNPs for TSMR analysis (Supplementary Table S2).

### 3.2 TSMR analysis results of cM and atrial fibrillation, or cM and warfarin

**Figure 2:**
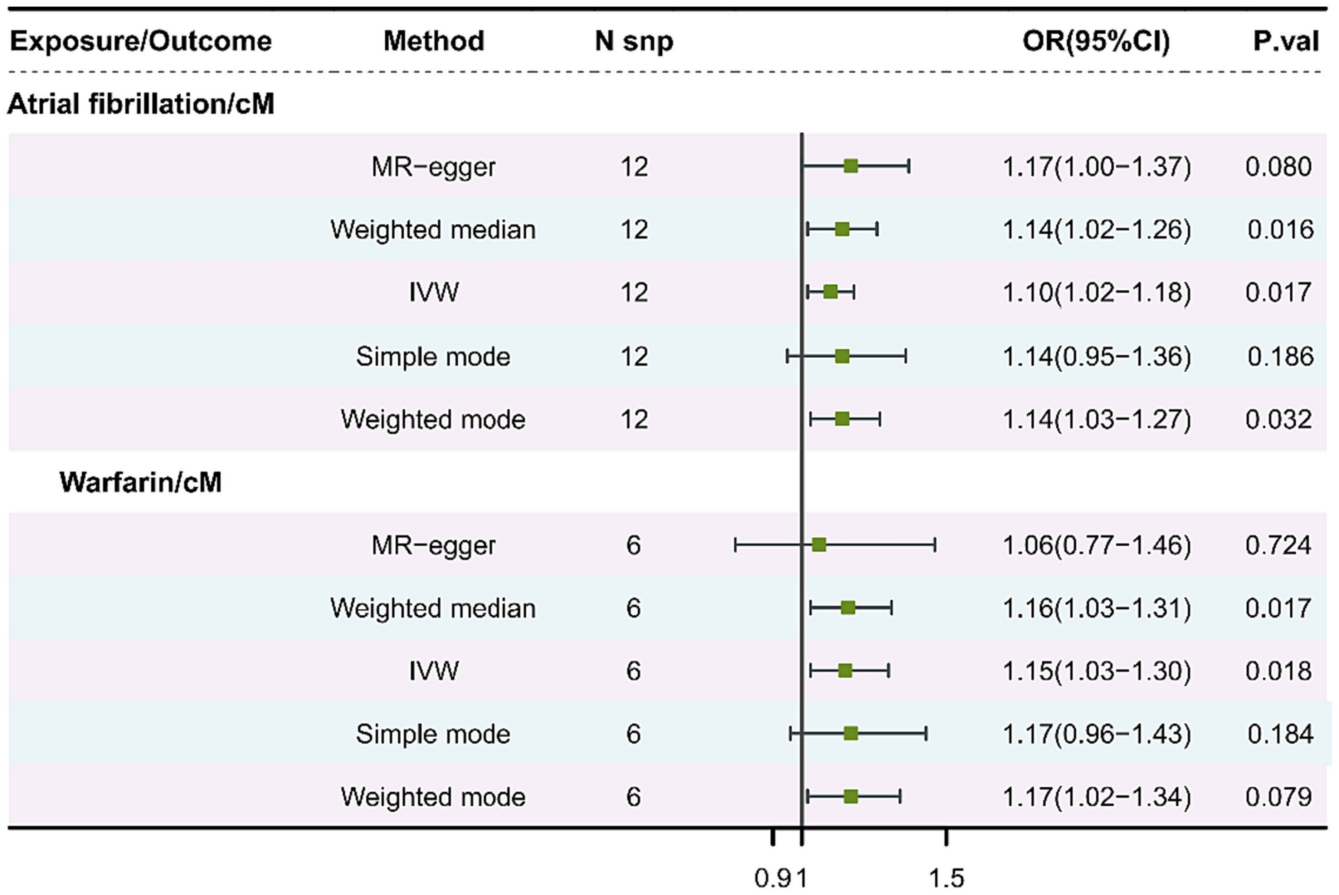
MR analysis of cM and atrial fibrillation, or cM and warfarin. cM, Cutaneous melanoma; MR, Mendelian randomization; SNP, Single nucleotide polymorphism; OR, Odds ratio; CI, Confidence interval; and IVW, Inverse-variance weighting.

In Figure 2, the IVW model outcomes for cM and atrial fibrillation showed that atrial fibrillation was positively linked with cM risk (*p* = 0.0387, OR = 1.10, 95%CI = 1.02–1.118). Meanwhile, The IVW model findings showed that warfarin was positively linked with cM risk in the warfarin and cM analysis results (*p* = 0.0175, OR = 1.15, 95%CI = 1.03–1.30). The above results show that atrial fibrillation and warfarin are the bad factors of cM.

### 3.3 Sensitive analysis of cM and atrial fibrillation, or cM and warfarin

Sensitivity studies were undertaken to test the pleiotropy and heterogeneity of the analyses. Table 2 displays the findings of the sensitivity analysis. As the table shows, there was no evidence to establish SNP pleiotropy in the MR-Egger regression analysis (*p* = 0.3379 > 0.05, *p* = 0.6154 > 0.05). When utilizing Cochran’s *Q* to assess for heterogeneity, it was discovered that tool SNPs in atrial fibrillation and cM analysis were not heterogeneous (*p* = 0.4383 > 0.05), and SNPs in warfarin and cM analysis were not heterogeneous too (*p* = 0.2339 > 0.05). It should be pointed out that when utilizing the MR-PRESSO test, the results produced during each regression test are different since the test requires simulation and considering the dependent variable has a high number of values, and the data given are the average of numerous results. Following that in the analysis of atrial fibrillation and cM, instrumental SNPs showed no horizontal pleiotropy (*p* = 0.419 > 0.05) and no outlier SNPs. Furthermore, in the analysis of warfarin and cM, instrumental SNPs did not demonstrate horizontal pleiotropy (*p* = 0.362 > 0.05) and did not contain outlier SNPs.

**Table 2:** Sensitive analysis of cM and atrial fibrillation, or cM and warfarin.

Exposure/outcome	Horizontal pleiotropy					Heterogeneity	
	MR-Egger regression			MR-PRESSO		Cochran’s *Q*	*p* value
	Egger intercept	SE	*p* value	Global test *p* value	Outliers		
Atrial fibrillation/cM	−0.0002	0.0002	0.3779	0.419	NULL	10.0248	0.4383
Warfarin/cM	0.0002	0.0004	0.6154	0.362	NULL	6.8258	0.2339

**Figure 3:**
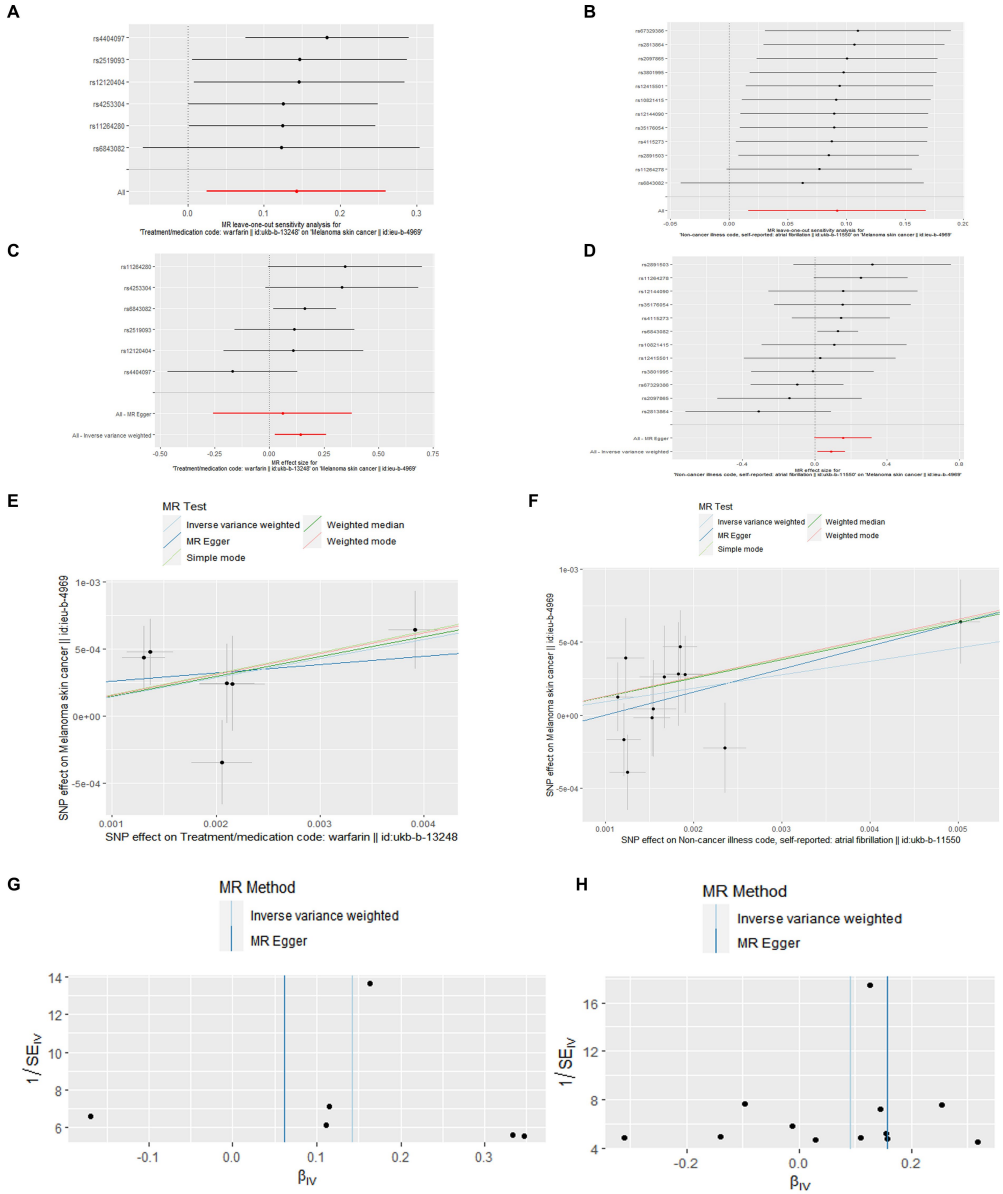
Leave-one-out plot **(A,B)**, forest plot **(C,D)**, scatter plot **(E,F)**, and funnel plot **(G,H)** of the causal effect of atrial fibrillation/warfarin on cM risk.

As for the outcomes of Figure 3, in the leave-one-out analysis, we can see that removing a single SNP does not have much effect on the overall result, and that no single SNP on the surface has a significant impact on the overall result. In a word, the leave–one–out sensitivity analysis confirmed the above conclusion. In addition, through the funnel plot in Figure 3, we can see that the points representing causal effects are symmetrical left and right, which shows that causal effects are unlikely to be affected by potential bias. However, the funnel plot of cM and warfarin is still needing forward study to verification.

## 4 Discussion

There exists a certain relationship between cM and atrial fibrillation, or cM and warfarin in many studies, but the causal relationship remains unclear. Understanding the interplay between cM with atrial fibrillation/warfarin could potentially guide the consideration of atrial fibrillation risks in the use of immune checkpoint inhibitors (26), BRAF (Serine/threonine protein kinase B-raf)/MEK (mitogen-activated protein kinase; MAPKK) inhibitors (12), and other targeted therapies for late-stage metastatic cM patients, as well as raise awareness of cM risks in atrial fibrillation patients, or cM risks in using warfarin for preventing thrombosis of cM patients with atrial fibrillation.

In our study, the TSMR method and genetic variation as instrumental variables are used to provide random evidence for the causal relationship between cM and atrial fibrillation. Therefore, our study can answer whether atrial fibrillation is a factor leading to cM from a new perspective. Besides, our research has some advantages. Its main advantage lies in relying on the data from large-scale and whole-genome GWAS, which provides a strong and reliable association of cM and atrial fibrillation SNPs and avoids the potential use of weak tools. Instead of using these SNPs (cM, atrial fibrillation) for statistical analysis, it reduces the possibility of confounding factors; on the contrary, it improves the reliability of the results. Our findings show a positive correlation between atrial fibrillation and the risk of cM (*p* = 0.0387, OR = 1.10, 95% CI = 1.02–1.118), suggesting that atrial fibrillation is a “positive factor” for cM. Combining genetic factors and UV radiation is the main inducing factor of cM (1), which can warn patients with atrial fibrillation to reduce ultraviolet radiation. This reduces the risk of cM. In addition, patients with atrial fibrillation should be treated to reduce the risk of cM, which may be related to changes in the internal environment, cell degeneration and necrosis, DNA breakage, mutation, and other negative changes that lead to tumor occurrence (5, 27). In this project, TSMR was used for the first time to reveal the relationship between cM and atrial fibrillation, which has important practical meaning.

Atrial fibrillation can also cause a series of adverse events, such as the risk of thromboembolism (13). According to the literature, the risk of stroke in patients with malignant tumors is higher than that in the general population, especially in patients with malignant tumors, whether or not they are complicated with atrial fibrillation (13, 14). At present, it is still controversial whether the risk of thromboembolism in patients with tumor complicated with atrial fibrillation is increased. However, it is inevitably used to prevent thromboembolism under the condition of meeting the no contraindication of using anticoagulants (warfarin represented) (14). Therefore, our research has designed the research on the causal relationship between warfarin and cM. Interestingly, our results show that there is a weak positive correlation between warfarin and the risk of cM (*p* = 0.0175, OR = 1.15, 95% CI = 1.03–1.30). In this respect, there is little support in the relevant literature. For example, Ambrus (28) found that even if patients with atrial fibrillation with new tumor take anticoagulants such as warfarin, the risk of stroke is still higher than that of the general population without tumor. The specific mechanism may not be clear. However, it is opposed to the literature that warfarin can inhibit the proliferation of tumor cells in basic cell experiments and be beneficial to preventing the recurrence of malignant melanoma (15, 29, 30). Our results suggest that warfarin is a positive factor of cM, which may be related to the side effect of warfarin, which can cause skin tissue death (necrosis) (31). In patients with cM accompanied with atrial fibrillation, the skin may always tolerate the influence of warfarin to prevent thrombosis caused by atrial fibrillation, which may lead to changes in skin cells of patients with atrial fibrillation and induce cM. In addition, cachexia, atrial fibrillation, warfarin, and other factors lead to the disorder of blood system function (32), which may affect the structure and function of skin tissue. In sum, warfarin and cM need further support and verification in basic and clinical research.

Finally, our study has some limitations. The first is that the samples studied were all European, so whether our findings can be extrapolated to other ethnic groups requires further research. Second, there are still some unclear confounders that can bias the experimental results. Finally, our study also lacks more data on cM, warfarin, and atrial fibrillation, and there is no causal relationship between warfarin and cM.

## Data availability statement

The original contributions presented in the study are included in the article/Supplementary material; further inquiries can be directed to the corresponding authors.

## Ethics statement

The manuscript presents research on animals that do not require ethical approval for their study.

## Author contributions

WX: Writing – original draft, Methodology, Investigation, Formal analysis, Data curation, Conceptualization. ZQ: Conceptualization, Writing – original draft, Visualization, Validation, Formal analysis, Data curation. HY: Writing – original draft, Formal analysis, Data curation. ZW: Writing – original draft, Formal analysis, Data curation. JY: Writing – original draft, Formal analysis, Data curation. CX: Writing – original draft, Formal analysis, Data curation. LS: Writing – review & editing, Supervision, Resources, Project administration. CJ: Writing – review & editing, Supervision, Resources, Project administration.

## Footnotes

^1^ http://gwas-api.mrcieu.ac.uk/

^2^ https://www.ukbiobank.ac.uk/

^3^ https://gwas.mrcieu.ac.uk/

^4^ http://www.phenoscanner.medschl.cam.ac.uk/
